# Computational approaches for discovering significant microRNAs, microRNA-mRNA regulatory pathways, and therapeutic protein targets in endometrial cancer

**DOI:** 10.3389/fgene.2022.1105173

**Published:** 2023-01-10

**Authors:** Ghada Ajabnoor, Fai Alsubhi, Thoraia Shinawi, Wisam Habhab, Walaa F. Albaqami, Hussain S. Alqahtani, Hisham Nasief, Nabeel Bondagji, Ramu Elango, Noor Ahmad Shaik, Babajan Banaganapalli

**Affiliations:** ^1^ Department of Clinical Biochemistry, Faculty of Medicine, King Abdulaziz University, Jeddah, Saudi Arabia; ^2^ Department of Genetic Medicine, Faculty of Medicine, King Abdulaziz University, Jeddah, Saudi Arabia; ^3^ Department of Medical Laboratory Technology, Faculty of Applied Medical Sciences, King Abdulaziz University, Jeddah, Saudi Arabia; ^4^ Princess Al-Jawhara Al-Brahim Center of Excellence in Research of Hereditary Disorders, King Abdulaziz University, Jeddah, Saudi Arabia; ^5^ Department of Science, Prince Sultan Military College of Health Sciences, Dhahran, Saudi Arabia; ^6^ Department of Clinical Laboratory Sciences, Prince Sultan Military College of Health Sciences, Dammam, Saudi Arabia; ^7^ Department of Obstetrics and Gynecology, Faculty of Medicine, King Abdulaziz University, Jeddah, Saudi Arabia

**Keywords:** endometrial cancer, microRNA, gene expression, PPI, cancer drug targets

## Abstract

Endometrial cancer (EC) is a urogenital cancer affecting millions of post-menopausal women, globally. This study aims to identify key miRNAs, target genes, and drug targets associated with EC metastasis. The global miRNA and mRNA expression datasets of endometrial tissue biopsies (24 tumors +3 healthy tissues for mRNA and 18 tumor +4 healthy tissues for miRNAs), were extensively analyzed by mapping of DEGs, DEMi, biological pathway enrichment, miRNA-mRNA networking, drug target identification, and survival curve output for differentially expressed genes. Our results reveal the dysregulated expression of 26 miRNAs and their 66 target genes involved in focal adhesions, p53 signaling pathway, ECM-receptor interaction, Hedgehog signaling pathway, fat digestion and absorption, glioma as well as retinol metabolism involved in cell growth, migration, and proliferation of endometrial cancer cells. The subsequent miRNA-mRNA network and expression status analysis have narrowed down to 2 hub miRNAs (hsa-mir-200a, hsa-mir-429) and 6 hub genes (*PTCH1, FOSB, PDGFRA, CCND2, ABL1, ALDH1A1*). Further investigations with different systems biology methods have prioritized *ALDH1A1, ABL1 and CCND2* as potential genes involved in endometrial cancer metastasis owing to their high mutation load and expression status. Interestingly, overexpression of *PTCH1*, *ABL1* and *FOSB* genes are reported to be associated with a low survival rate among cancer patients. The upregulated hsa-mir-200a-b is associated with the decreased expression of the *PTCH1, CCND2, PDGFRA, FOSB* and *ABL1* genes in endometrial cancer tissue while hsa-mir-429 is correlated with the decreased expression of the *ALDH1A1* gene, besides some antibodies, PROTACs and inhibitory molecules. In conclusion, this study identified key miRNAs (hsa-mir-200a, hsa-mir-429) and target genes *ALDH1A1*, *ABL1* and *CCND2* as potential biomarkers for metastatic endometrial cancers from large-scale gene expression data using systems biology approaches.

## Introduction

Endometrial cancer (EC) is a urogenital cancer mostly affecting post-menopausal women. Globally, it is ranked as the fourth most prevalent cancer affecting women and the 15th most frequently diagnosed type of cancer overall (Gu et al., 2021; World Cancer Research Fund International, 2020). The most common symptoms of EC are abnormal vaginal bleeding, pelvic pain, unexplained weight loss, and difficulty urinating. EC is basically divided into types 1 and 2. Type 1 tumors (endometrioid adenocarcinomas) show slow growth, less aggression, and are caused by excessive estrogen. Whereas type-2 tumors (non-endometrioid endometrial cancer) are fast-growing, more aggressive and are not caused by excessive estrogen. The key risk factors implicated in EC include excessive estrogen, obesity, diabetes mellitus, late menopause, early menarche, and nulliparity (American Cancer Society, 2022). Early detection of EC by physical examination, medical imaging, and biopsies is necessary to offer early treatment for better clinical outcomes. More than 75% of the patients are diagnosed at stage 1, which has a higher survival rate (<1% of all cancer deaths, 2% of cancer deaths in women) (Gu et al., 2021; World Cancer Research Fund International, 2020). Standard management of EC involves surgical removal of the uterus, ovaries, and fallopian tubes but sometimes may also involve radiation therapy, chemotherapy, and hormonal therapy (Crosbie et al., 2022).

In general, genetic factors account for 2%–10% of endometrial cancer. Twin studies estimated that the heritability of EC is about 27%–52%. The large-scale Genome Wide Association Studies (GWAS) have identified many common genetic variants influencing EC risk, which were mapped to 16 loci on different chromosomes with their approximate relative risk in family members is as high as 7%. The most reported genes with mutation in EC are *P53*, *CCND2*, and *KRAS*, along with the defective DNA mismatch repair genes. Furthermore, more genes were discovered, and their roles became clearer with the help of bioinformatic approaches. And these studies revealed the involvement of several genetic mutations in endometrial cells, including tumor suppressor genes and oncogenes such as *PTEN*, and *PIK3CA*, respectively (Mucci et al., 2016; Johnatty et al., 2017; Cheng et al., 2016). However, molecular pathogenesis of EC is highly complex due to the genetic, and environmental interactions, where the later ones alter the expression of many genes connected to cell growth, metabolism, and cell division in endometrium (Galaal et al., 2014; Dörk et al., 2020).

There has been an emerging interest in the role of miRNAs in altering the gene expression landscape in cancer pathogenesis. Recent research has linked increased and unregulated expression of microRNAs to carcinogenesis in endometrial cancer. A comparison between endometrial cancer tissue and normal tissue shows that the expression of certain microRNAs is higher in the cancerous than in the healthy tissues. Examples are mir-103, mir-106a, mir-107, mir-181a, mir-185, mir-205, mir-210, mir-423, mir-429 and mir-449. On the contrary, the expression of number of other microRNAs is lower in cancerous cells. These include mir-30c, mir-99b, mir-193, mir-152, mir-193b, mir-204, mir-221 along with miRlet7e. Also, miRNAs are responsible for high expression of *SRY-*related high mobility group box4 (*SOX4*) in endometrial cancer, as well as loss of DNA mismatch repair (*DNA-MMR*) genes, *PTEN* and many other key genes that play an important role in endometrial carcinogenesis (Boren et al., 2008; Chung et al., 2009; Wang Q et al., 2020).

Over the recent years, advanced computational methods have shown their strength in identifying tissue based molecular biomarkers by analyzing the complex global gene expression data of different human diseases. Identifying these key gene expression signatures may hold the key to monitor cancer progression, which would eventually allow early clinical intervention and to develop personalized cancer therapy. The dynamic molecular landscape underlying the hyperproliferation and transformation of normal endometrium into endometrial cancer can be better understood by global profiling of genes and miRNAs. But the information on the role of differentially expressed miRNAs (DEMiRs) and their target genes (DEGs) between normal and endometrial cancer tissues is still not well explored. Therefore, this study aims to study the complex and dynamic molecular interactions among miRNA, target genes by employing robust bioinformatic gene network analysis and advanced statistical tools.

## Materials and methods

### Datasets collection for endometrial Cancer’s DEGs and DEMiRs

The endometrial cancer gene expression datasets were downloaded from National Center for Bioinformatics-Gene Expression Omnibus (NCBI-GEO) as well as EMBL-EBI Array express database, using the key terms such as endometrial cancer, genetics, mRNA, and miRNAs. Then based on the expression array, data quality, sample types and sample numbers two data sets were selected. The first dataset (GSE115810) consists of mRNA expression profiles of 24 tissue samples of different grades of endometrial cancer (G1: 7 specimens; G2: 11 specimens; and G3: 6 specimens) compared to three control endometrium tissues, generated on GPL96 Affymetrix Human Genome U133A Array platform (Hermyt et al., 2019). The second dataset (GSE35794) consists of miRNA expression profiles of 22 samples (18 endometrioid endometrial cancer, 4 normal endometrium controls) analyzed on Agilent-021827 Human miRNA Microarray V3 (miRBase release 12.0 miRNA ID version). Since, we utilized the secondary datasets of gene expression, we don’t have any control on the sample size, analysis methods and statistical power.

### Identification of DEGs and microRNAs in endometrial cancer tissues

To identify dysregulation of specific genes in endometrial cancer, we carried out a differential expression analysis of the miRNA and mRNA profiles of endometrial tissues. We used the NCBI webtool GEO2R (https://www.ncbi.nlm.nih.gov/geo/geo2r/) to identify differentially expressed genes (DEGs) as well as differentially expressed microRNAs (**DEMiRs**) in the test datasets. The GEO2R webtool was used to perform comparative analysis on microarray expression data sets using the GEO query and Limma R programs from Bioconductor software. Limma, a R Bioconductor package, was used to identify the genes that demonstrated 1.5-fold changes (FC) and miRNAs that demonstrated 3 FC with adjusted *p*-values < .05. The expression pattern of these mRNAs and miRNAs was depicted graphically as a volcano plot with median mean difference.

### Identification of potential DEmiRs target genes

Initially, the miRNA IDs of the DEmiRNA have been used to search for their possible target genes in the MiRDB webserver (http://mirdb.org/mirdb/mining.html). The exclusion criteria were based on ruling out any target genes with less than 70 prediction scores and any miRNAs having more than 2,000 predicted genome-based targets to reduce the false positive rate. After that, we check the shared DEGs to assess the overlap with potential DEmiRNA target genes. Then, using the miRDB data as a reference, an inverse correlation analysis was conducted between the expression levels of miRNA and mRNA to find the miRNA-target gene pairs. By using the Venny 2.1.0-BioinfoGP tool, we identified the inverse correlation by merging the upregulated miRNA target genes with downregulated DEGS and *vice versa*. The mathematical formula used to find the inverse correlation between miRNA and mRNA levels are shown below, in which “n” represents the correlation coefficient, “X” represents DEGs, and “Y” represents DEMs.

$$r=n\left(\sum xy\right)-\sum x\sum y$$

$$\sqrt{\left[n*\left(\sum x2-\left(\sum x\right)2\right)\right]*\left[n*\left(\sum y2-\left(\sum y\right)2\right)\right]}$$

### Construction of miRNA-target gene transcriptome network

The miRNet webserver (http://www.mirnet.ca) was used for building the functional interactome network of endometrial cancer genes and miRNA. The input options for this webserver consist of Entrez/Ensemble gene ID and miRbase ID for DEGs and DemiRs, respectively. This program generates a transcription network with network properties like degree of centrality, closeness centrality as well as betweenness centrality. Genes and miRNAs with the highest centrality scores were selected from the network as hub genes, and then KEGG pathway, functional enrichment, and transcription factor association analysis were performed.

### The miRNA-target gene functional analysis

The hub genes detected in the miRNA-target gene pairs were further investigated at this phase to assess their expression status in endometrial tissues, drug tractability and mutational load in endometrial tumors.

### Identification of drug tractability of hub genes

The open target platform (http://www.targetvalidation.org) was utilized to investigate the hub genes from the miRNA-target gene network. The query gene ID was used as an input. Then, data regarding phenotype association characteristics (*p*=<0.05), known drug information (target disease, mode of action, clinical trial phase) and drug tractability predictions (small molecule, antibodies, PROTAC along with other modalities) were generated for the query gene.

### Identification of mutation load of hub genes

The mutational load of hub genes detected from miRNA-target gene network was assessed with the help of cBioPortal (http://www.cbioportal.org/) webserver. The oncoprint summary in this webserver visualizes the genetic changes of the queried genes in the form of heatmaps with z-score values after giving the gene ID and cancer type as input data. Moreover, Log2 Odds Ratio (OR) values were used to calculate the mutation pattern of hub gene pairs in endometrial cancer.

### The expression and prognostic significance analysis of hub genes across endometrial cancers

The GEPIA2 (http://gepia2.cancer-pku.cn/) webserver was used to examine the expression status of query hub genes in normal and cancer tissues to analyze their disease-free survival correlation. The gene whose log2 fold change was equal to or more than one was considered significant at a *p* < .01.

### Hub gene expression in endometrial cancer tissues using immunohistochemistry

The expression status of hub genes in cell lines and tissues was determined using the Human Protein Atlas (https://www.proteinatlas.org) database. With a *p*-value <0.05, a log rank test was used to find a correlation between the expression status of hub genes in both the endometrial cancer and normal tissues. With the input of query gene or protein name, this database will provide the immunocytochemistry/immunofluorescence (ICC-IF) data regarding subcellular location as well as expression of the candidate protein (protein-transcripts per million, pTPM). Additionally, immunohistochemistry data was also analyzed to determine the expression of query genes. The visual appearance of the image, detector grain settings used in its acquisition, the intensity of primary antibody staining between normal and tumor tissues, were considered to score the expression as negative, weak, moderate, or strong grade.

### Phenotype impact analysis of hub genes on endometrial cancer phenotypes in knockout mouse models

The Mouse Genome Informatics database (http://www.informatics.jax.org/) was used to investigate the role of query DEGs in endometrial cancer phenotypes in knockout mouse models. HGNC gene symbols were provided as input to this webserver. The output was in the form of graphical representation indicating the phenotypes affected by the knock down of candidate gene.

## Results

### Identification of differentially expressed target genes and miRNAs

**FIGURE 1:**
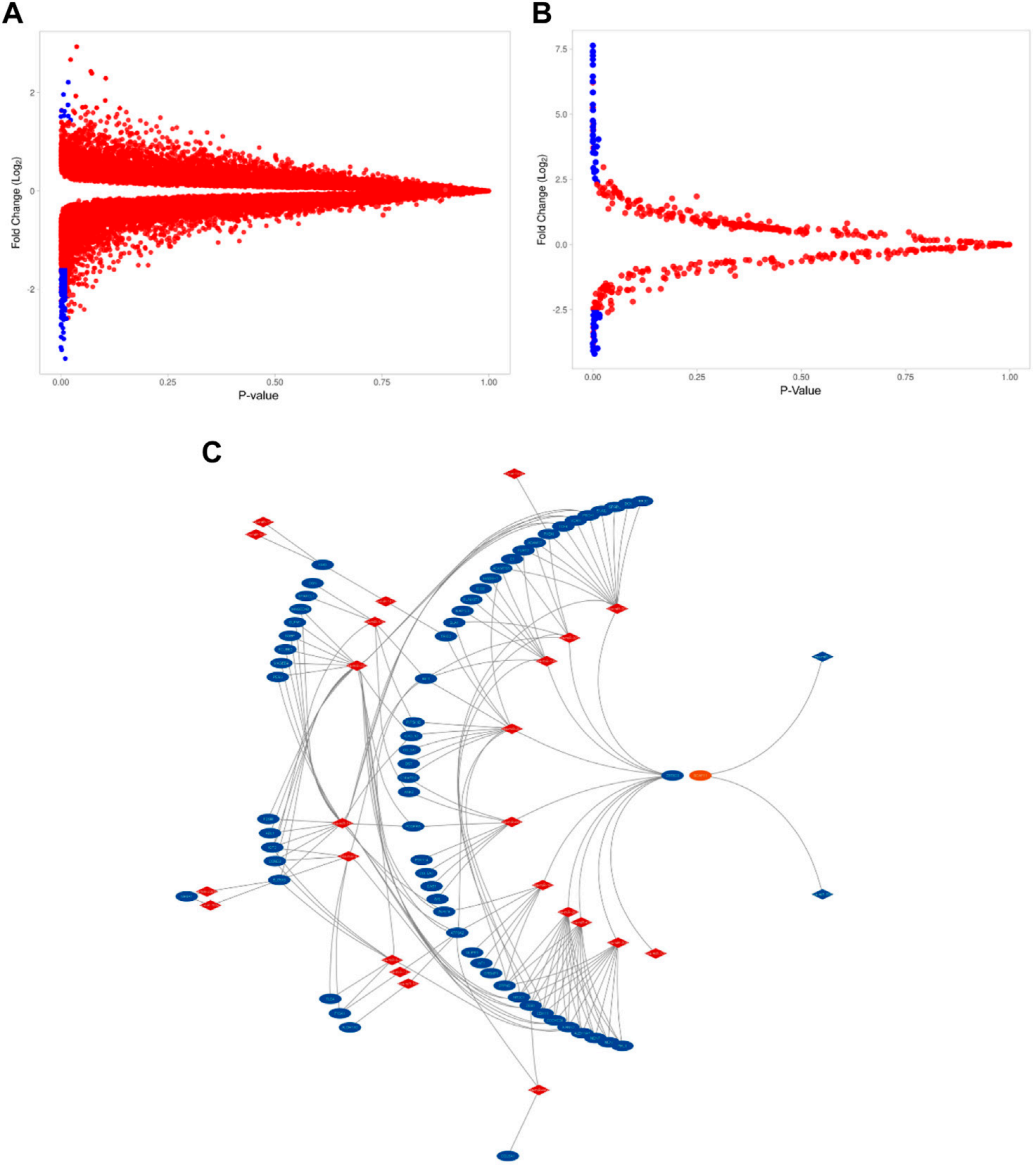
The expression level of DEGs and DEmiRNAs. **(A)**. Volcano plots of GSE115810 showing the DEGs as blue dots. (DEGs with FC > 2.5, *p*-value <0.05) **(B)**. Volcano plots of GSE35794 showing the DEmi as blue dots (Demi with FC>>2.5, *p*-value <0.05). **(C)**. Differentially expressed miRNA (in red) and their target DEGs (in blue) interaction network.

The GSE115810 dataset has the expression data of 21,156 genes with 22,283 probes. In total, 192 genes were differentially expressed (log2FC +/−1.5) between EC and normal tissues. Of these DEGs, 29 (15%) were upregulated, and 163 (85%) were downregulated (*p*=< 0.05). The miRNAs corresponding to 961 probes were identified from the GSE35794 dataset. Endometrial cancers show a total of 43 DEmiRNAs (log2 = 3 folds). These DEmiRNAs, 29 (67.5%) were up- and 14 (32.5%) down-regulated when compared to normal endometrial tissues (*p*=<0.05). Figures 1A, B reveal the expression levels of DEGs and DEmiRNAs respectively in both normal and cancerous states. Figure 1C show the interaction network of DEGs and DEmiRNAs. Tables 1 summarizes the top six upregulated and downregulated DEGs and DEmiRNA, respectively.

**TABLE 1:** The top six upregulated DEGs and DEmiRNAs in endometrial cancer tissues.

Datasets	ID	*p*	T	B	logFC	Gene
**GSE115810**	204623_at	0.0368201	2.192344	−3.71965	2.9339558	*TFF3*
	204846_at	0.0226967	2.411272	−3.32777	2.6669008	*CP*
	212531_at	0.0699633	1.88409	−4.22729	2.4332492	*LCN2*
	204051_s_at	0.0102181	−2.754032	−2.66974	−3.4142217	*SFRP4*
	219791_s_at	0.0000345	−4.919383	2.13964	−3.1751096	*HAND2-AS1*
	207016_s_at	0.0067949	−2.922448	−2.32938	−3.0086929	*ALDH1A2*
**GSE35794**	hsa-miR-200b	3.15E-07	6.280183	6.59	7.633409	hsa-miR-200b
	hsa-miR-205	1.31E-05	5.05709	3.17	7.413916	hsa-miR-205
	hsa-miR-200a	1.85E-07	6.455611	7.079	7.382017	hsa-miR-200a
	hsa-miR-133b	3.93E-03	−3.08527	−2.032	−4.18593	hsa-miR-133b
	hsa-miR-873	4.25E-04	−3.887906	−0.021	−4.066755	hsa-miR-873
	hsa-miR-1	1.10E-02	−2.683293	−2.943	−3.981862	hsa-miR-1

Footnotes: T-moderated t-statistic, *p*- *p*-Value, B- B statistics or log-odds that the gene is differentially expressed. LogFC- Log2-fold change between EC, and healthy tissues.

### miRNA-target gene mapping

At a cutoff score of 70, the miRDB webserver estimated that of 43 DEmiRNA, 35 are affecting the expression of 14,411 target genes. Of the 14,411 target genes, 113 were overlapping with 192 DEGs detected in endometrial cancer tissues. In addition, the inverse correlation analysis between DEmiRNA and DEGs have further narrowed down the DEGs to 66 and DEmiRNA to 25 (Figure 1C). Majority of the downregulated genes (65/66) were targeted by 24 upregulated DEmiRNA. On the other hand, only one upregulated DEG that is targeted by two downregulated DEmiRNA. Table 2 highlights the top miRNA-target gene pairs detected in endometrial cancer tissues.

**TABLE 2:** The DEmiRNA-target gene pairs in endometrial cancer tissues.

DEmiRNA	#	List of miRNAs	Target gene	Target score	Log Fc. miRNA	Log Fc. DEGs
Up-Reg miRNA	1	hsa-miR-1228	*ATP8A2*	85	4.383286	−1.7159646
			*F13A1*	79	4.383286	−2.2489688
	2	hsa-miR-375	*ZBTB20*	92	4.397115	−1.8853867
	3	hsa-miR-182	*ATP8A2*	79	4.516506	−1.7159646
			*ALDH1A2*	77	4.516506	−3.01
Down-Reg miRNA	1	hsa-miR-1	*SCAF11*	84	−3.981862	1.6274417
	2	hsa-miR-133b	*SCAF11*	72	−4.18593	1.6274417

Footnotes: LogFC: log fold changes. **# =** serial number.

### Pathway enrichment of miRNA-Target genes

Using the KEGG pathways, we performed the network enrichment for 66 target genes (65 downregulated genes and one upregulated gene) and 25 miRNAs at the *p*-value threshold of < 0.05 which are summarized in Table 3. All the downregulated genes were enriched in endometrial cancer along with other types of cancers, like focal adhesions, *p53* signaling pathway, ECM-receptor interaction, Hedgehog signaling pathway, fat digestion and absorption, glioma as well as retinol metabolism. However, the up-regulated genes were not enriching any KEG pathways.

**TABLE 3:** The functional enrichment of downregulated genes in KEGG pathways.

Pathway	Number of genes	List of genes	*p*-value
Focal adhesion	7	*IGF1, CCND2, MYLK, COL3A1, PDGFRA, COL5A2, COL5A1*	2.57E-07
p53 signaling pathway	3	*CCND2, PERP*	3.22E-06
		*IGF1*	
Pathways in cancers	6	*RUNX1T1, PDGFRA, ABL1, PTCH1, IGF1, FOS*	1.14E-05
ECM-receptor interaction	3	*COL5A1, COL5A2, COL3A1*	1.01E-04
Hedgehog signaling pathway	2	*PTCH1, GAS1*	1.45E-05
Fat digestion and absorption	1	*PLPP1, ALDH1A1, ALDH1A2, PDGFRA, IGF1*	1.45E-05
Retinol metabolism	2		1.45E-05
Glioma	2		1.45E-05

*p*-value = < .05 Significant.

### Analysis of the miRNA-target gene interaction network

**FIGURE 2:**
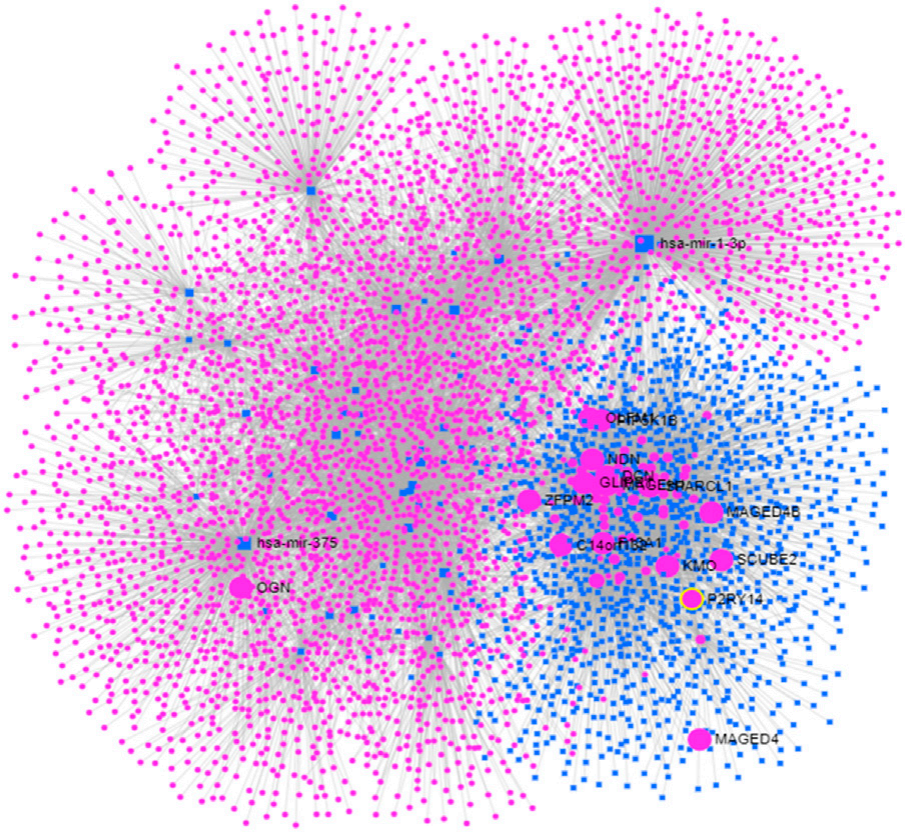
The miRNA-genes network of endometrail cancer based on significant global differential expression profile of miRNAs and mRNAs. Pink circles indicate genes while blue squares refers to miRNA.

Molecular networks show the physical interactions between protein partners. They are essential for the fundamental molecular systems in cellular function but are frequently altered in disease states. In the miRNA-mRNA network, there were 5,374 nodes and 10,322 edges for the 66 target genes and 26 miRNAs (Figure 2). Based on our stringent filtration criteria for the network parameters (miRNA with > 50 centrality; > 85888.082), we identified the hubs genes; 18 target genes and five miRNAs (hsa-mir-429, hsa-mir-200a, hsa-mir-200b-5p, hsa-mir-141-5p and hsa-mir-143-5p). Moreover, 3 miRNAs (hsa-mir-200b-5p, hsa-mir-141-5p, hsa-mir-143-5p) do not have any target genes. Therefore, they were eliminated from further analysis, and we ended up with two upregulated DEmiRNA (hsa-mir-200a and hsa-mir-429), targeting 5 and 1 downregulated genes (*PTCH1, FOSB, PDGFRA, CCND2, ABL1; ALDH1A1*) respectively.

Furthermore, functional enrichment revealed that these miRNAs are involved in carbohydrate metabolism, stem cell regulation, type II pneumocyte differentiation, cell adhesions, histone modifications (26662986), cell motility, and migration. Supplementary Table S1 summarizes the contribution of each miRNA to the mentioned functions. In addition, transcription factors analysis for each of the five miRNAs were performed. Supplementary Table S2 shows each miRNA with its corresponding transcription factor.

### System biology validation of endometrial cancer hub genes

We used multiple system biology approaches to assess the functional validity of the six hub genes (together with the inversely regulated miRNAs listed above) in endometrial cancer.

### The molecular tractability of hub genes

In order to assess the genotype-phenotype association score for the six hub genes (6/18; 33.3%) from the topological analysis, we used the Open Target Validation Platform. All of them show a 0.01 association score, with the exception of the *PTCH1* gene shows 0.00 association scores. In addition, the tractability information was available for the 5 genes except for the *FOSB* gene. To illustrate, *ALDH1A1*, *ABL1* and *CCND2* genes were tractable by small molecules and also targeted by Proteolysis Targeting Chimeras (PROTACs) While *PTCH1* and *PDGFRA* genes were tractable by small molecules, PROTACs along with antibody molecules. Considerably, *ABL1* is a molecular target for both Imatinib (treating chronic myeloid leukemia) and Mesylate which is in phase I clinical trial for endometrial cancer. Also, *PDGFRA* is targeted by Nintedanib and Dovitinib in phase II clinical trial and by Vatalanib and Cediranib in phase I clinical trial. This information is summarized in Table 4.

**TABLE 4:** Open target platform phenotype association and Target trackability assessment of the endometrial cancer hub genes.

Gene	Geno-pheno association	Phenotype	Known drugs	Action	Clinical trial phase	Tractability predictions			
						^1^Small molecule	^2^ Antibody	^3^PROTAC	^4^Other modalities (enzymes, peptide, oligonucleotides
*ALDH1A1*	0.01	High in glioblastoma multiforme (0.17)	-	-	-	4, 5, 8	-	6,7,8	-
*ABL1*	0.31	High in chronic myelogenous leukemia (0.82)	IMATINIB MESYLATE	Inhibitor	I	1,4,5,6,8	-	4,5,6,8	-
			IMATINIB	Inhibitor	I				
			DASATINIB	Inhibitor	0				
*FOSB*	0.03	High in heel bone mineral density (0.17)	-	-	-	-	-	-	-
*PDGFRA*	0.26	High in Gastrointestinal stromal tumor (0.84)	NINTEDANIB	Inhibitor	II	1,4,5,8	1,4,5,7	5,6,7,8	1
			DOVITINIB	Inhibitor	II				
			VATALANIB	Inhibitor	I				
			CEDIRANIB	Inhibitor	I				
*CCND2*	0.25	High in colorectal cancer (0.47)	-	-	-	5,8	-	5,6	-
*PTCH1*	0.00	High in basal cell carcinoma (0.70)	-	-	-	4	5,6,7	5,6	-

Footnotes: ^1^Small Molecules: 1. Approved Drug, 2 Advanced Clinical, 3. Phase 1 Clinical, 4. Structure with Ligand, 5. High-Quality Ligand, 6. High-Quality Pocket, 7. Med-Quality Pocket, 8. Druggable Family.^2^Antibody: 1. Approved Drug, 2. Advanced Clinical, 3. Phase 1 Clinical, 4. UniProt loc high conf, 5. GO CC, high conf, 6. UniProt loc med conf, 7. UniProt SigP or TMHMM, 8. GO CC, med conf, 9 Human Protein Atlas loc. ^3^PROTAC: 1. Approved Drug, 2. Advanced Clinical, 3. Phase 1 Clinical, 4. Literature, 5. UniProt Ubiquitination, 6. Database Ubiquitination, 7.Half-life Data, 8. Small Molecule Binder. ^4^ Other Modalities: 1. Approved Drug, 2. Advanced Clinical, 3. Phase 1 Clinical; *p*-value = < .05 Significant.

### The mutation load assessment of hub genes

**FIGURE 3:**
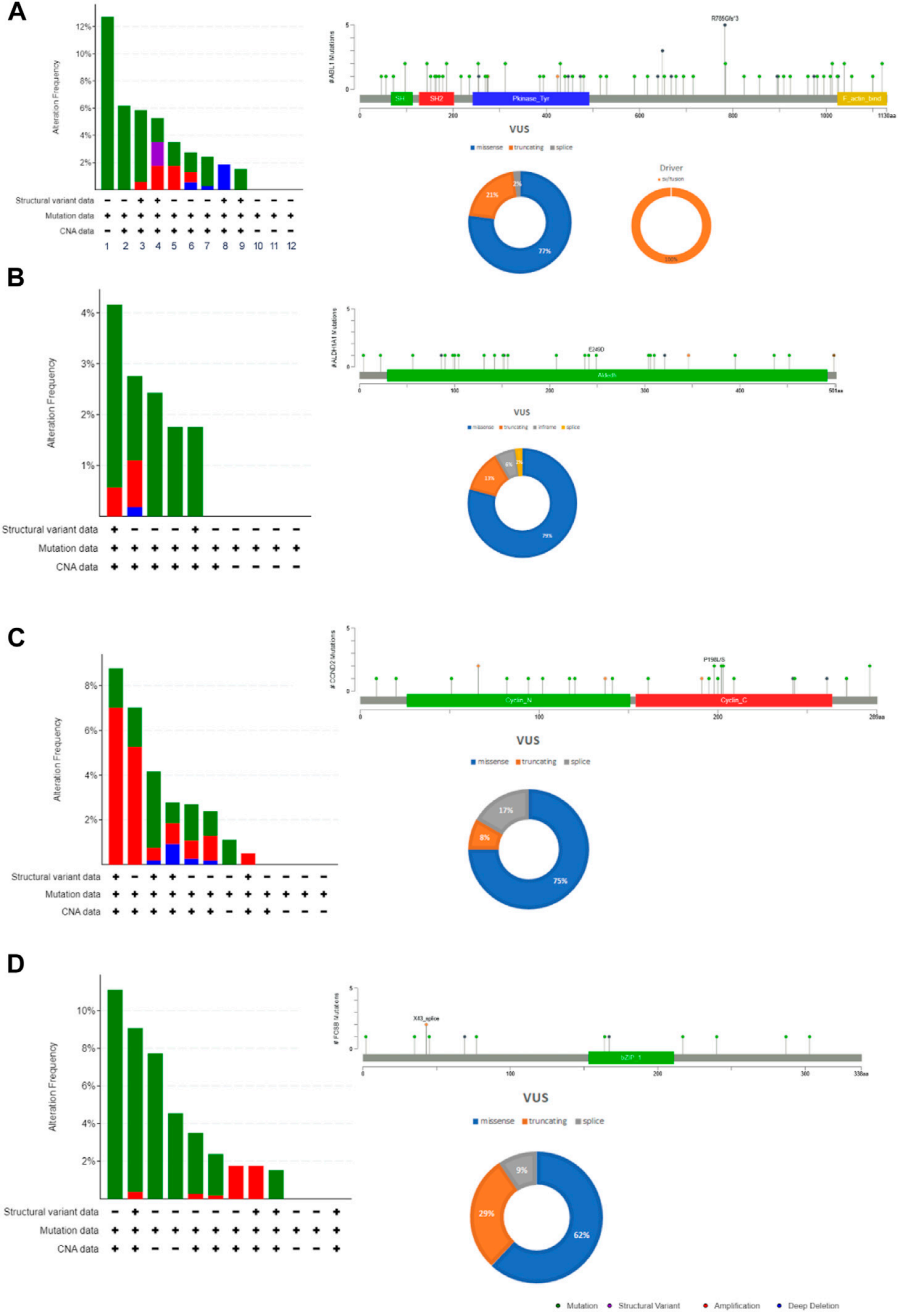
The mutation load of hub genes in endometrial cancers. **(A)**. *ABL1*, **(B)**. *ALDH1A1*, **(C)**. *CCND2* **(D)**. *FOSB*. The distribution and frequency of genetic changes in endometrial cancer’s hub genes. The lollipop plot and pie chart determine the type of mutation and localization of the mutation in a protein.

**FIGURE 4:**
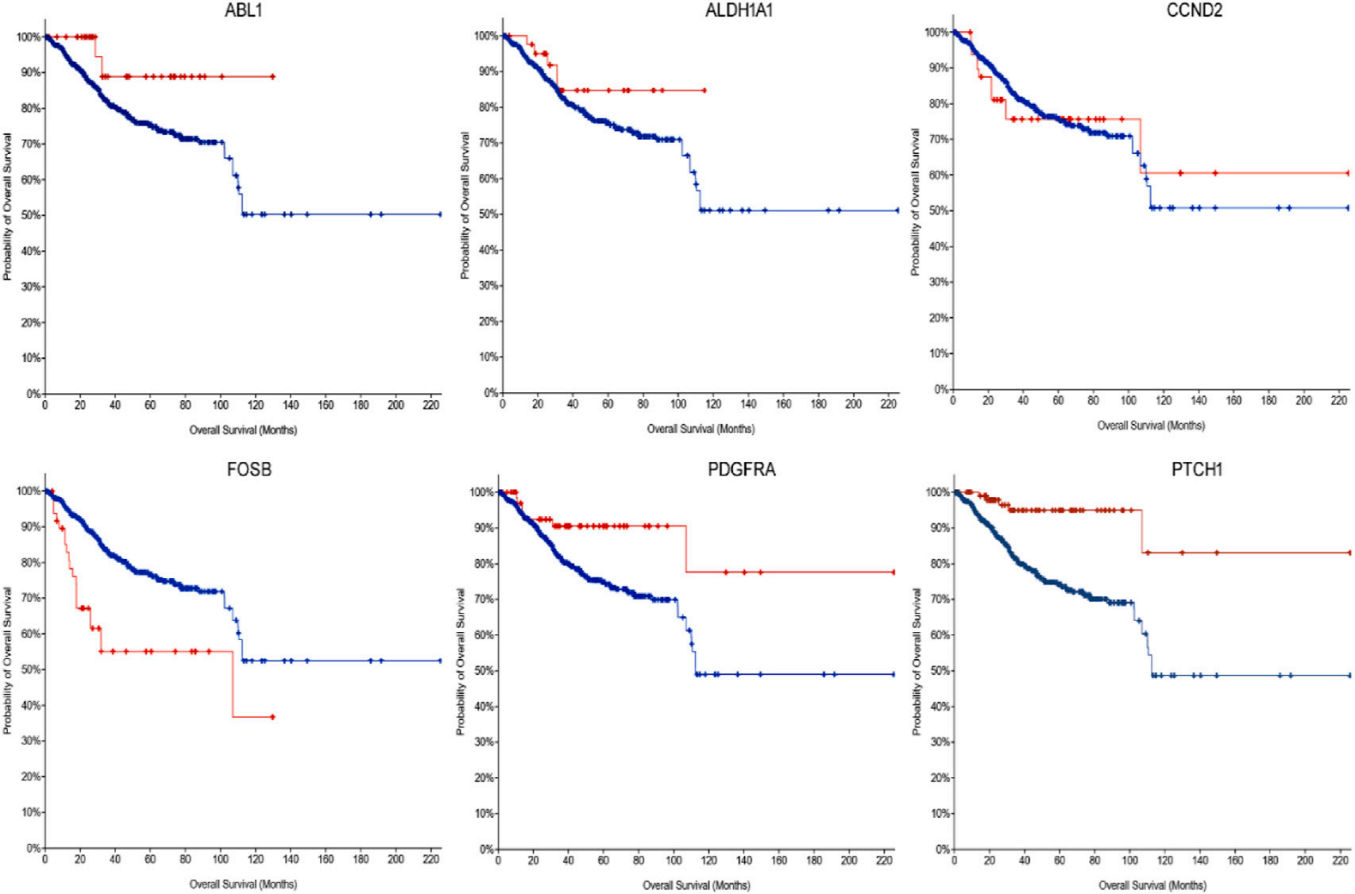
The Kaplan-Meier survival curve for the dysregulated hub gene as prognostic indicator in endometrial cancers (disease survival in months). *ABL1* Log rank *p* = 0.0199, *ALDH1A1* Log rank *p* = 0.197, *CCND2* Log rank *p* = 0.923, *FOSB* Log rank *p* = 3.722e-5, *PDGFRA* Log rank *p* = 9.494e-3, and *PTCH1* Log rank *p* = 6.914e-5.

The cBioportal for Cancer Genomic Analysis has confirmed that all the hub genes show diverse types of genetic alterations including missense, truncating, frameshift, amplifications, deep deletion, splice mutations, etc. They are displayed in Figures 3A–D and Supplementary Figures S1A–C. Interestingly, *PTCH1* shows the highest mutation rate in 7% (127/1867; 7%) of cancers, followed by *PDGFRA* (89/1867; 5%), *ABL1* (Figure 4) (68/1867; 4%), *FOSB* (56/1,678, 3%), *ALDH1A1* and *CCND2* (48/1,678; 2.9%), (55/1867; 2.9%). Interestingly, mutations were found in the functional domain of all six genes suggesting their potential contribution in the development of endometrial cancers. Furthermore, the mutations in all six genes reportedly co-occur with each other (OR is 3; *p* < 0.001) (Table 5). The survival curve analysis shows that, out of the six genes; 1 gene shows significant result in which the high expression level of *FOSB* is associated with a low survival rate. Figure 4 depicts the correlation between the dysregulation of these genes and the survival status of patient.

**TABLE 5:** The co-occurrence of mutations in the hub genes of endometrial cancer.

A	B	Neither	A Not B	B Not A	Both	Log2 odds Ratio	*p*-value	q-Value	Tendency
*PDGFRA*	*PTCH1*	1,258	41	75	47	>3	<0.001	<0.001	Co-occurrence
*PDGFRA*	*CCND2*	1,308	64	25	24	>3	<0.001	<0.001	Co-occurrence
*ALDH1A1*	*PDGFRA*	1,118	21	63	22	>3	<0.001	<0.001	Co-occurrence
*ALDH1A1*	*PTCH1*	1,092	19	89	24	>3	<0.001	<0.001	Co-occurrence
*CCND2*	*PTCH1*	1,272	27	100	22	>3	<0.001	<0.001	Co-occurrence
*ABL1*	*PTCH1*	1,260	39	101	21	2.748	<0.001	<0.001	Co-occurrence
*ALDH1A1*	*CCND2*	1,145	31	36	12	>3	<0.001	<0.001	Co-occurrence
*FOSB*	*CCND2*	1,142	34	37	11	>3	<0.001	<0.001	Co-occurrence
*FOSB*	*PDGFRA*	1,106	33	73	12	2.462	<0.001	<0.001	Co-occurrence
*FOSB*	*PTCH1*	1,078	33	101	12	1.956	<0.001	<0.001	Co-occurrence
*ABL1*	*PDGFRA*	1,284	49	77	11	1.904	<0.001	0.001	Co-occurrence
*ABL1*	*CCND2*	1,319	53	42	7	2.052	0.004	0.004	Co-occurrence
*ALDH1A1*	*FOSB*	1,141	38	40	5	1.908	0.018	0.021	Co-occurrence
*ABL1*	*FOSB*	1,127	52	40	5	1.438	0.054	0.058	Co-occurrence
*ALDH1A1*	*ABL1*	1,127	40	54	3	0.646	0.324	0.324	Co-occurrence

### Confirmation of the hub gene expression status in endometrial cancers

**FIGURE 5:**
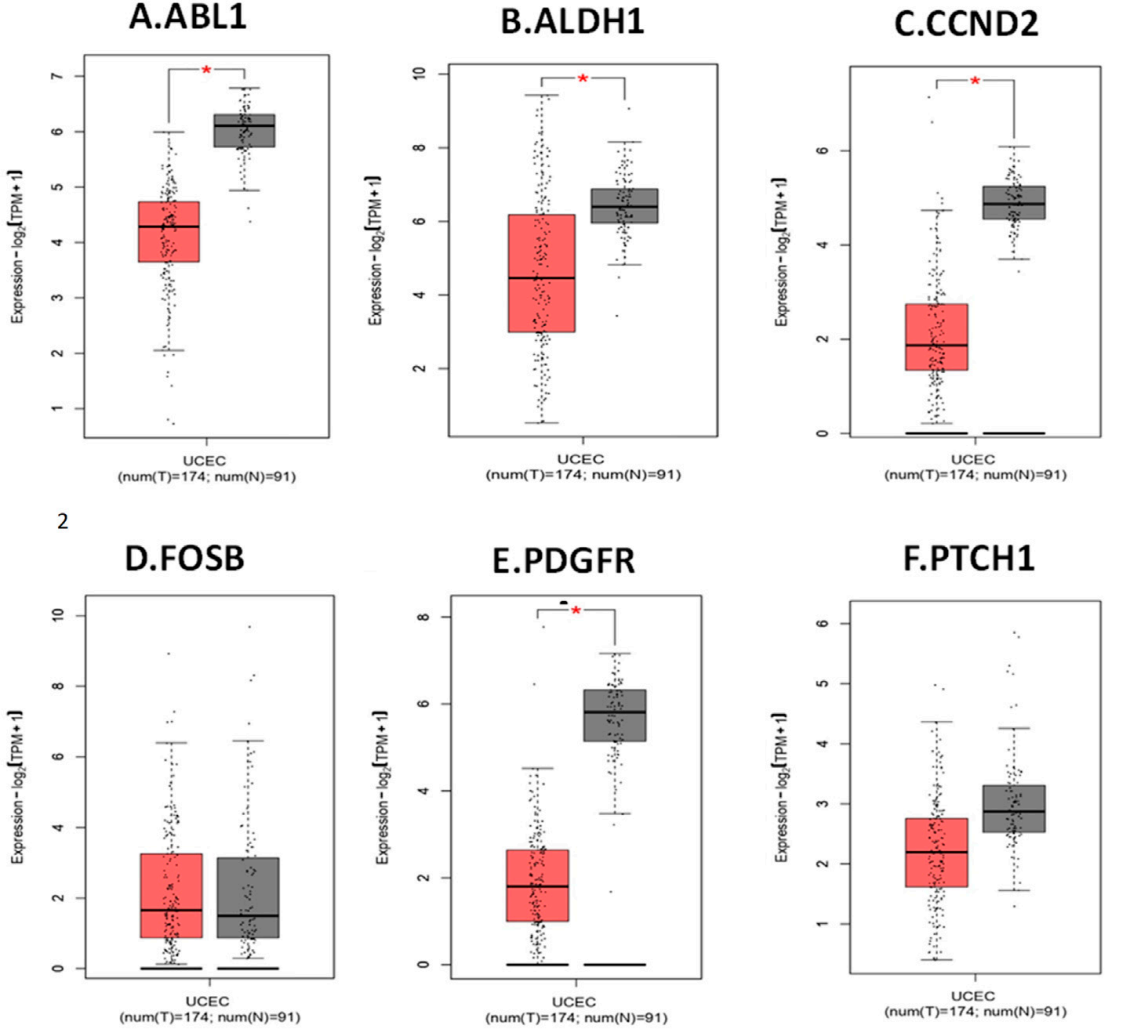
Expression profile of hub genes in endometrial cancer compared to normal tissue (from GEPIA2). **(A).** *ABL1*. **(B).** *ALDH1A1*. **(C).** *CCND2*. **(D).** *FOSB*. **(E).** *PDGFRA.* **(F).** *PTCH1*. The Y-axis determine the mean value of log2 (TPM +1; Log2FC > 1; *p*-value <0.01). X-axis determine the samples. The red box represents EC samples, while the gray box represents normal tissues. Each dot represents an individual sample in the category.

Assessment of the expression levels of the six hub genes in Gene Expression Profiling Interactive Analysis (GEPIA) showed 3 genes (*ABL1, ALDH1A1 and PDGFRA*) show significant upregulated expression in endometrial cancer, confirming that these genes play a significant role in endometrial carcinogenesis. Figure 5 depicts the expression boxplot of the six hub genes.

### Analysis of immunocytochemistry and immunofluorescence

**FIGURE 6:**
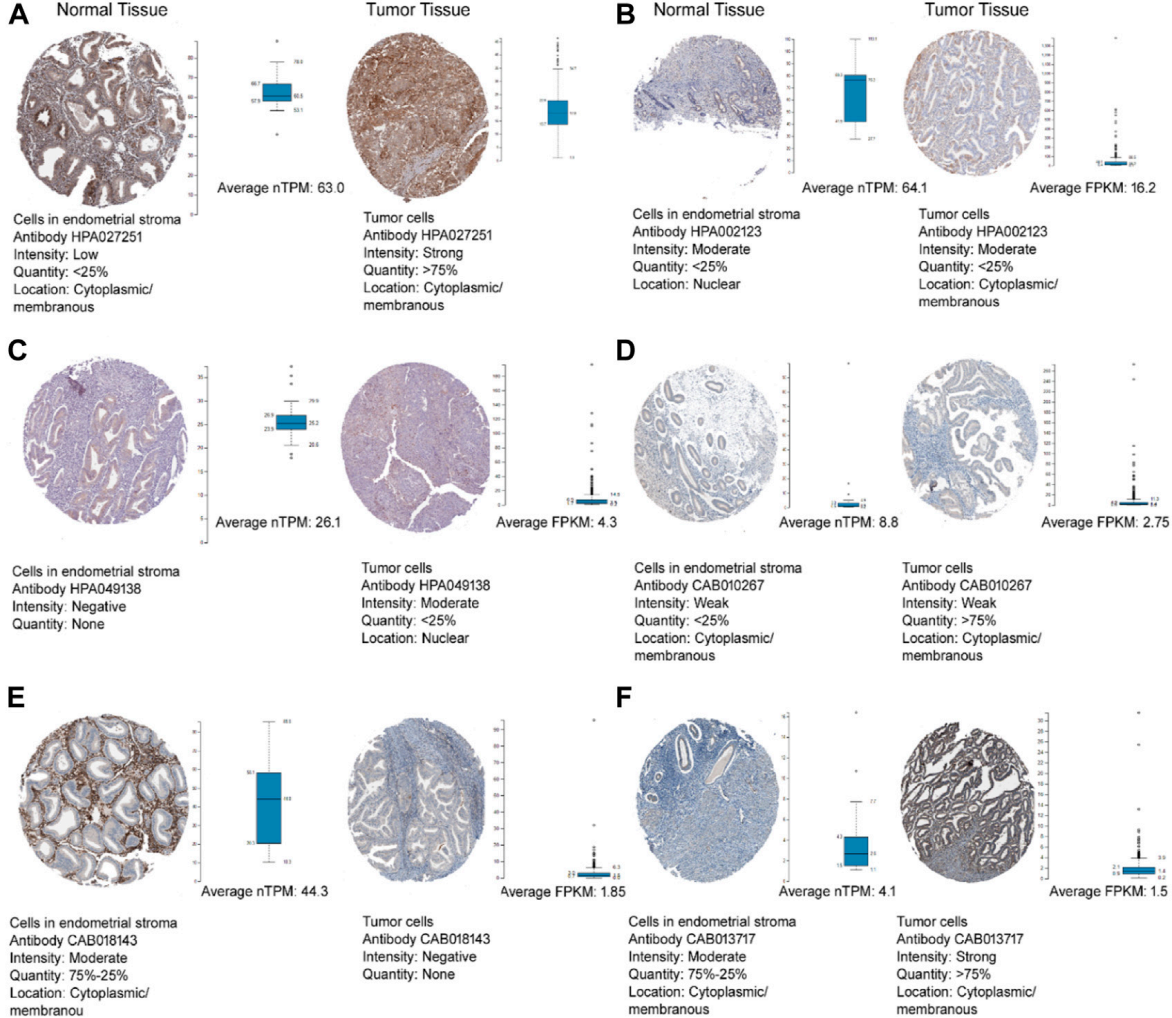
The Human Protein Atlas immunohistochemistry of hub genes in endometrial cancer and normal endometrial tissues (magnification of 4X10) **(A)** *ABL1*, **(B)** *ALDH1A1*, **(C)** *CCND2*, **(D)** *FOSB*, **(E)** *PDGFRA*, and **(F)** *PTCH1*. Immunohistochemistry figure represented the antibody, staining intensity and subcellular localization. The whisker box plot determines the hub genes TPM and FPKM values in normal and tumour tissues.

**FIGURE 7:**
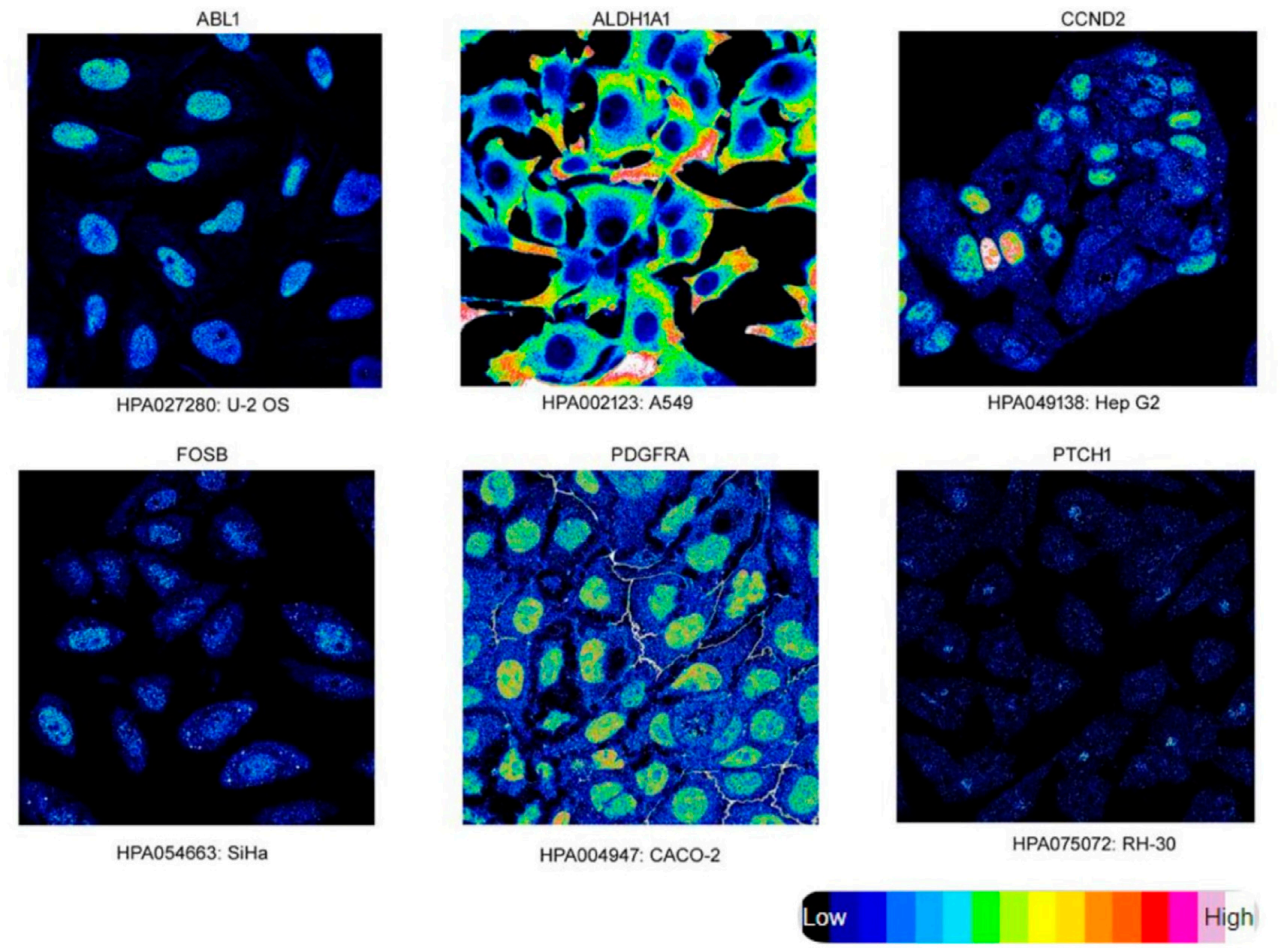
Immunofluorescence Staining. Localization and expression of hub genes in endometrial tissue. Endometrial Cancer hub genes (target gene) expression (intensity) in various cell lines, subcellular staining represents target proteins with intensity using different antibodies.

Figure 6 depicts the protein expression of the six hub genes in endometrial cancer tissues. *PTCH1* gene showed the highest expression level in cancer tissue when we compared it to normal endometrium tissue with medium to high intensity. The subcellular localization of hub genes in various cell compartments in several human cancer cell lines can be determined by HPA indirect immunofluorescence analysis. The expression data was expressed in the form of FPKM (fragments per kilobase of exon model per million reads mapped). Most of the hub genes were found to be concentrated in the nucleoplasm, *ABL1* (16.2 FPKM), *PDGFRA* (1.5 FPKM), *FOSB* (1.4 FPKM), and *CCND2* (3.5 FPKM), while *PTCH1* in Golgi apparatus (1.5 FPKM) and *ALDH1A1* in the cytosol (16.2 FPKM). We were able to map protein expression patterns of hub genes and pinpoint their sub-cellular locations in individual cells. Based on this analysis, we confirm the dysregulated protein expression levels of the six hub genes in endometrial malignancies. Figure 7 shows the expression of these hub genes in their major subcellular locations.

### Analysis of mouse studies by knockout models

We examined the phenotype impact of all the six query hub genes in knockout mouse based on the Mouse Genome Informatic database. Absence or defective *PDGFRA, CCND2* and *PTCH1* genes in mouse results in abnormal reproductive system phenotypes.

### Concordance analysis

**FIGURE 8:**
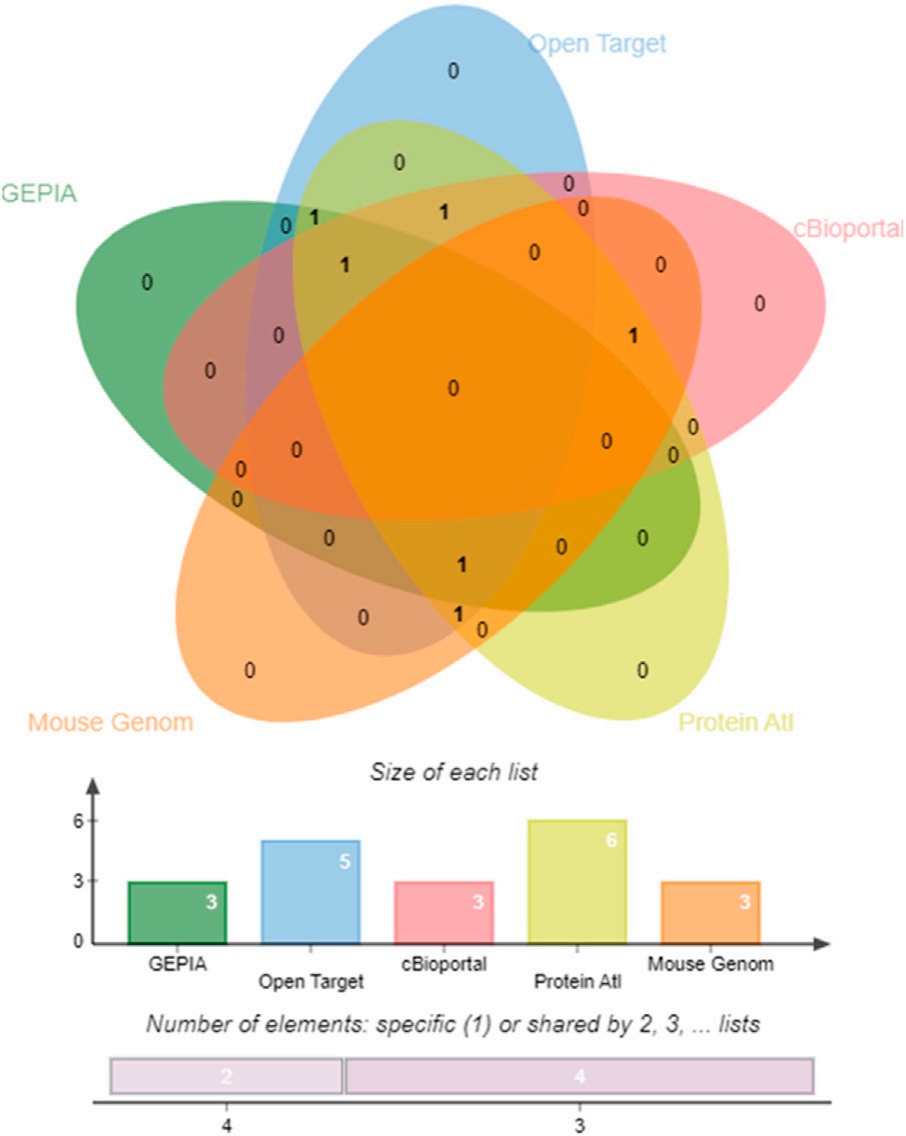
Edwards’ Venn diagram. Prioritizing the impactful hub genes through the system biology methodology of the pooled significant outcome from 5 functional element analyses. A Venn diagram depicting the overlap of six genes. Horizontal Bar Graph represent the number of hub genes supported by individual functional elements, The Vertical Bar Diagram represent the evidence from four elements for two genes, and three elements for four genes.

Concordance analysis of the six hub genes and two miRNAs using several system biology paradigms is depicted in Figure 8. We consider the genes with significant results in at least three validation tools to play a very key role in endometrial carcinogenesis. *ABL1* and *PDGFRA* are confirmed by four tools. The remaining 4 genes (*PTCH1*, *ALDH1A1*, *ALDH1A1*, and *CCND2*) are confirmed by three tools.

## Discussion

The clinical presentation of endometrial cancer is quite variable, ranging from a localized, non-aggressive to highly aggressive, rapidly spreading, and invasive lethal cancer. These variations are due to different intrinsic factors such as genetic alteration, complex gene expression patterns, cell lineage and histological subtypes interacting with variety of environmental factors including exposures to mutagens or carcinogens (Bell and Ellenson, 2019). Identification of these factors at an early stage may have positive impact on developing molecular diagnosis and targeted therapies of endometrial cancer with minimum toxicity. Molecular subtyping of primary endometrial cancers using different bioinformatics tools and analysis methods were reported earlier with many limitations (Banaganapalli et al., 2020; Besso et al., 2020; Kim et al., 2020; Wang G et al., 2020). However, these studies used different study design, sampling method, statistical measures, and validation approaches. Even though the number of publications aimed at studying the effect of miRNAs is increasing, understanding of miRNAs role in the carcinogenesis process and their effect on gene expression is lacking (He et al., 2015; Liu Y et al., 2020; Miao et al., 2021).

In this study we characterized the molecular connections between key genes and microRNAs involved in endometrial metastasis using publicly accessible expression datasets from several endometrial tissues. We identified 66 differently expressed genes in endometrial cancer tissues (65 downregulated and 1 was upregulated). The KEGG pathway identifies no enriched pathways that were associated with the upregulated gene. However, pathways of ECM-receptor interaction, *p53* signaling and cancer are highlighted by the enrichment of key downregulated genes. The extracellular matrix (ECM) of the female reproductive tract undergoes extensive structural remodeling on a monthly basis, through its components modification, accumulation or degradation. By directly encouraging cellular transformation and metastasis, abnormal ECM has an impact on the cancer progression. Remarkably, stromal cell activity is also deregulated by ECM abnormalities which promotes inflammation as well as angiogenesis resulting in the formation of cancer microenvironments. The ECM-receptor interaction signal pathway has also been identified as a contributor to cancer progression with low survival rate in many gynecological malignancies including endometrial cancer, breast cancer and ovarian cancer (Bao et al., 2019; Liu et al., 2021; Yadav et al., 2020). In this study, the expression of *COL3A1*, *COL5A1* and *COL5A2* were downregulated in EC, are of the collagen family, and they interact with elements of the extracellular matrix (ECM) including matrix metalloproteinases, tyrosine kinase receptors, integrins, and signaling pathways to affect the behavior and activity of cancer cells. Studies found that the outcome/survival rate of EC patients with low *COL5A2* expression was considerably lower than patients with high *COL5A2* expression. Despite that, farther studies are needed to clarify the exact mechanism so we can understand the prognostic and therapeutic values of such dysregulation (Jabłońska-Trypuć et al., 2016; Zhu et al., 2020).

In addition, prognosis in EC was predicted by the *P53/P21* signaling pathway, which was linked to patients’ age, stages, and mortality. Since *P53* is responsible for the regulation of the cell growth and proliferation, dysfunction in *P53* signaling pathway was associated with elevation of cyclin D1 and proliferating cell nuclear antigen (PCNA) which promote higher proliferation in endometrial tissue (Liu Q et al., 2020).

We also discovered miRNA changes that target genes controlling the transformation of normal endometrial tissue to aggressive malignant ones. High estrogen level, overweight, diabetes mellitus and other human disorders that are known to raise the risk of endometrial cancer can be better understood by finding hub genes, with the highest degree of centrality in the key module (Zang et al., 2018). Therefore, when generating miRNA-mRNA functional network that are composed of 66 miRNA target genes and 26 miRNAs, we identified 18 hub target genes and 5 miRNAs with a high degree of centrality parameter (>50). Out of the hub miRNAs exclusively dysregulated in EC, only 2 (hsa-mir-200a, hsa-mir-429) were targeting 6 hub genes (*ALDH1A1, ABL1, FOSB, PDGFRA, CCND2, PTCH1*). Interestingly, all hub miRNAs were upregulated, and all the hub genes were downregulated.

Pathway analysis of the hubs (genes and miRNAs) revealed their engagement in a variety of biological processes such as cell division, migration, motility as well as regulation of stem cells in response to extracellular signals, histones modifications, carbohydrate metabolism and oncogenic transformation. Therefore, our findings are mostly consistent with earlier research that revealed differential regulation of key genes and microRNAs in the transformation of normal endometrial tissue to endometrial cancer (Lee et al., 2011; Wu et al., 2017; Huang et al., 2018; Guo et al., 2020; Zheng et al., 2021).

MicroRNAs and transcription factors (TFs) influence gene expression at the post-transcriptional and post-translational stages, respectively. Notably, the formation of a feed-forward loop (FFL) unit allows miRNAs and TFs to regulate each other as well as co-regulate a shared target gene, which further develops gene regulatory networks (Jiang et al., 2016). In this study, we found that both miR-429 and hsa-mir-200a-5p miRNAs regulate several TF-target genes such as *ZEB1*, *ZIB2* (*Smad-interacting protein 1*), *TGFB1* and *SP1* (specificity protein 1). In endometrial cancer cells, they regulate cytoskeleton remodeling, independent of the zinc finger E-box binding homeobox (ZEB)/E-cadherin axis, which in turn affects cell migration, and elongation plays a crucial role in specifying the cell phenotypes. However, overexpression of *ZEB2* is associated with oncogenic transformation and tumor metastasis in several cancer types such as endometrial, hepatocellular, and thyroid cancers (Liu et al., 2017). On the contrary, down regulation of *ZEB2* is associated with poor prognosis and showed an enhanced potency and invasiveness of colon cancer cells. So, its interaction with other genes in certain tumor/cancer microenvironment is still unclear and need further studies (Li et al., 2017). They also regulate *TGF-β* (transforming growth factor β) which in turn regulates the cellular pathways such as SMAD and ERK1/2 by positively influencing stress fiber production, cellular migration, survival and proliferation of cancer cells (Gregory et al., 2008; Wang et al., 2016; Xiong et al., 2016). *SP1*-TF plays a role in growth factors signaling, involved in immune response, chromatin remodeling and response to DNA damage (Klicka et al., 2021). Furthermore, we found *SIX1 (Sine Oculis Homeobox Homolog 1), GATA3* and *SMAD3*-TFs are regulated by hsa-mir-200a-5p miRNA. *SMAD3* suppresses tumor growth by preventing cell division and encouraging apoptosis. Additionally, *Smad3* controls transcriptional responses that contribute to metastasis and is necessary for TGF-beta-mediated immune suppression. Accordingly, depending on the kind of cell and clinical stage of the cancer, Smad3 works both as a negative and positive regulator of carcinogenesis by controlling various transcriptional responses (Millet and Zhang, 2007).

The comprehensive system biology validation of the six hub genes has shown that upregulated hsa-mir-200a-b is associated with the decreased expression of the *PTCH1, CCND2, PDGFRA, FOSB* and *ABL1* genes in endometrial cancer tissue. The upregulated hsa-mir-429 is correlated with the decreased expression of the *ALDH1A1* gene. By targeting inhibition of *PTEN* gene, miR-200a b can accelerate the growth of endometrial cancer cells (Wu et al., 2017). Of note, hsa-miR-429 is frequently increased in a variety of malignancies. It may act as an oncogene and associated with the decreased overall survival, increase in the cancerous cells growth and cell proliferation by inhibiting *CDKN2B* in cancers such EC (Banaganapalli et al., 2020; Liu et al., 2018). Also, it has been found that anti-miR-429 and mir200a-b could improve the cytotoxic activity of chemotherapy in EC’s patient (Lee et al., 2011; Banaganapalli et al., 2020). Based on this output, both miRNAs have the potential to be biomarkers and therapeutic target for EC.

Proteolysis Targeting Chimeras (PROTACs) can target all *ALDH1A1, ABL1, PDGFRA*, *CCND2* and *PTCH1* genes. Additionally, *PDGFRA* and *PTCH1* are targeted by antibody molecules. *ABL1* is targeted by Imatinib Mesylate inhibitor which, in a phase I clinical trial for endometrial cancer, while *PDGFRA* is targeted by Nintedanib, and Dovitinib inhibitors which are currently under phase II clinical trial and Vatalanib as well as Cediranib, inhibitors under phase I clinical trial.

There are a few limitations to this study. To establish non-invasive biomarkers for endometrial carcinogenesis, it is critical to map relevant miRNAs or genes in body fluids such as blood, urine, and vaginal discharges. This work has discovered a number of significant hub genes and miRNAs, which need to be further tested on a large pool of samples using validation methods such as real-time PCR, and functional biology assays. The number of clinical samples analyzed in this study is small. However, this limit is unreasonable given that we used secondary data retrieved from GEO and had no control over the study design. Although analyzing larger sample size, would result lower background noise and standard error of the effect fraction, but may not drastically change the study conclusion.

To sum up, the present study identifies 6 hub genes (*ALDH1A1, ABL1, FOSB, PDGFRA, CCND2, PTCH1*) along with two miRNAs (hsa-mir-200a, hsa-mir-429) significantly influencing multiple crucial cellular processes in endometrial cancer. This study demonstrates the efficacy of computational concepts, such as functional enrichment of biological pathways, and the construction of miRNA-mRNA and transcription factor gene networks, in the identification of endometrial cancer biomarkers from massive gene expression data. There is a correlation between tissue-based endometrial cancer indicators and cancer progression, which may allow for early clinical intervention and therapy. This study also lays the groundwork for future knowledge-driven functional analyses of the microRNAs and their gene targets in endometrial cancer.

## Data Availability

The datasets presented in this study can be found in online repositories. The names of the repository/repositories and accession number(s) can be found in the article/Supplementary Material.
