# Supplementary material for: Computational approaches for discovering significant microRNAs, microRNA-mRNA regulatory pathways, and therapeutic protein targets in endometrial cancer
Source: Front Genet. 2023 Jan 10;13:1105173. doi: 10.3389/fgene.2022.1105173 (PMC9872035; doi:10.3389/fgene.2022.1105173)
Supplement: Supplementary file 3 [file Table1.DOCX]

**Supplementary Table1**. miRNA contributions in function enrichment analysis.

| **Function** | **List of miRNA** | **Adj. P Value** |
| --- | --- | --- |
| Carbohydrate Metabolism | hsa-mir-200a-5p | 0.1768 |
|  | hsa-mir-429 |  |
| Regulation of Stem Cell | hsa-mir-200a-5p | 0.1768 |
|  | hsa-mir-429 |  |
| Type II Pneumocyte Differentiation | hsa-mir-200a-5p | 0.2318333 |
| Cell Adhesion | hsa-mir-200a-5p | 0.3614 |
| Histone Modifications (26662986) | hsa-mir-200a-5p | 0.3614 |
| Cell Migration | hsa-mir-200a-5p | 0.3614 |
| Cell Motility | hsa-mir-200a-5p | 0.3614 |

**Supplementary Table2.** miRNA with its corresponding transcription factors.

| **List of TF** | **List of miRNA** | **Adj.P Value** |
| --- | --- | --- |
| SIP1 | hsa-mir-200a-5p | 0.0005566 |
|  | hsa-mir-429 |  |
| ZEB2 | hsa-mir-200a-5p | 0.0005566 |
|  | hsa-mir-429 |  |
| SMAD3 | hsa-mir-200a-5p | 0.008172667 |
| TGFB1 | hsa-mir-200a-5p | 0.0088895 |
|  | hsa-mir-429 |  |
| ZEB1 | hsa-mir-200a-5p | 0.01886 |
|  | hsa-mir-429 |  |
| GATA3 | hsa-mir-200a-5p | 0.044735 |
| SIX1 | hsa-mir-200a-5p | 0.044735 |
| SP1 | hsa-mir-200a-5p | 0.044735 |
