# Supplementary figures and images for: Computational approaches for discovering significant microRNAs, microRNA-mRNA regulatory pathways, and therapeutic protein targets in endometrial cancer
Source: Front Genet. 2023 Jan 10;13:1105173. doi: 10.3389/fgene.2022.1105173 (PMC9872035; doi:10.3389/fgene.2022.1105173)

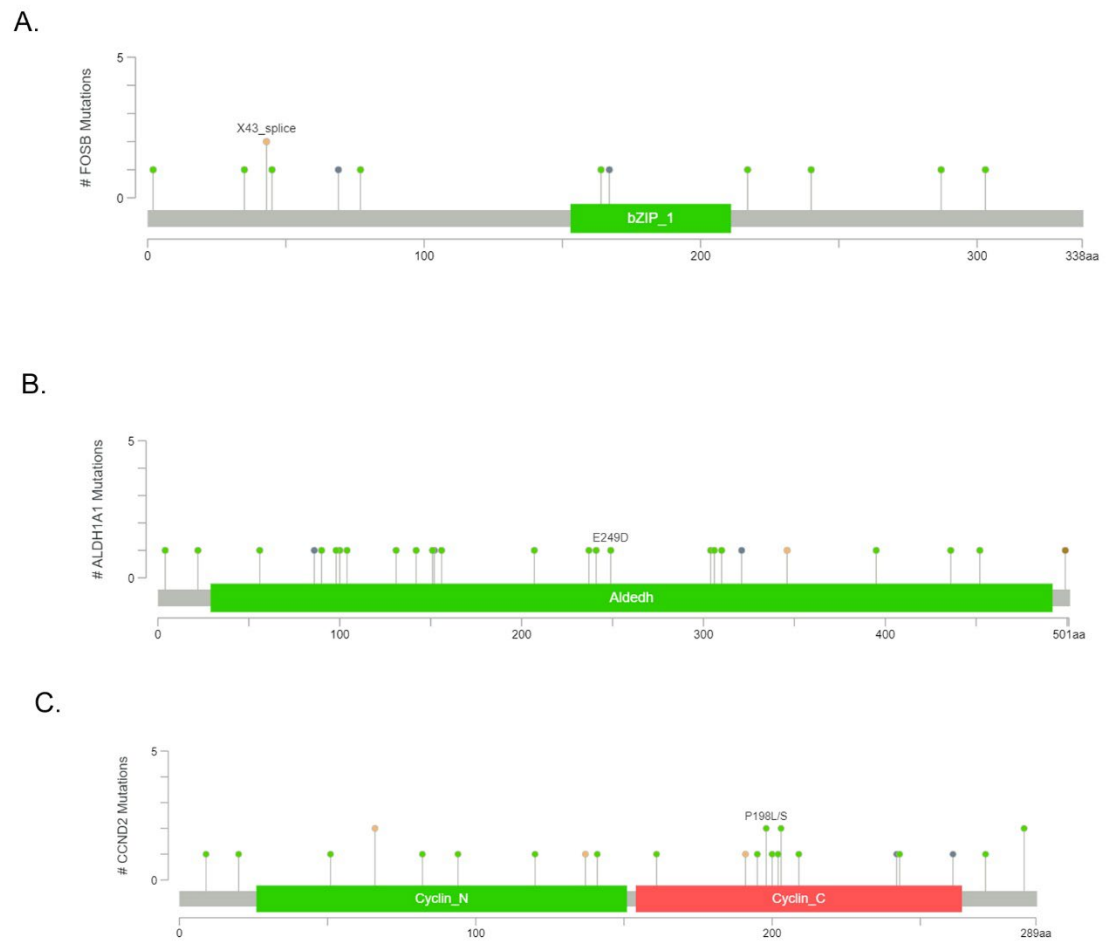

Supplementary Figure 1: Mutation distribution Lollipop graph of FOSB, ALDH1A1 and CCND2

Supplement: Supplementary file 6 [file Image1.pdf]
